# Double-plate compound osteosynthesis of a periprosthetic proximal tibial shaft fracture in an elderly patient with Paget's disease using PHILOS and LCP plates

**DOI:** 10.1016/j.tcr.2025.101275

**Published:** 2025-11-13

**Authors:** Melinda Schlink, Christian von der Lippe

**Affiliations:** a Department of Orthopaedics and Traumatology, Regional Hospital Herisau, Bahnhofstrasse 6, 9100, Herisau, Switzerland

**Keywords:** Paget's disease of the bone, Bone fragility, Periprosthetic fracture, Geriatric fractures, Compound osteosynthesis, PHILOS plate

## Abstract

**Introduction:**

Paget's disease of the bone (PDB) is characterized by abnormal bone remodeling and accelerated osteoclast activity, weakening bones and leading to deformities, fractures, or osteoarthrosis, commonly involving the tibia. Treating proximal tibial shaft fractures in PDB patients is challenging due to compromised bone quality and unique anatomical considerations. Despite ongoing research, clear guidelines or conclusive evidence on the optimal treatment strategies of these fractures remain limited.

**Case report:**

A 91-year-old male presented with severe right knee pain and inability to bear weight after falling down the stairs. Radiographs and computed tomography (CT) revealed a proximal tibial shaft fracture in a patient with prior total knee arthroplasty (TKA) and confirmed PDB. The fracture showed no prosthesis loosening but a deformity of the tibial plateau, making standard fixation unfeasible. The patient underwent an open reduction with double-plate compound osteosynthesis using a proximal humerus internal locking system (PHILOS ®, Depuy Synthes, Zuchwil, Switzerland) and a locking compression plate (LCP ®, Depuy Synthes, Zuchwil, Switzerland). At 6-weeks, he had a pain-free knee with full range of motion and was able to fully weight-bear. Radiographs showed stable fixation with no hardware loosening.

**Conclusion:**

In conclusion, double-plate compound osteosynthesis effectively managed a rare periprosthetic proximal tibial shaft fracture in a patient with PDB, achieving stable fixation and enabling immediate postoperative full weight-bearing and fast recovery.

## Introduction

PDB, first described by Sir James Paget in 1877 [1], is a chronic skeletal disorder caused by dysregulated bone remodeling. It is characterized by excessive bone resorption by osteoclasts and increased remodeling by osteoblasts, resulting in structurally weakened, sclerotic, and often deformed bone. These changes lead to bone pain, an increased risk of fractures, bowing deformities, and secondary osteoarthritis [2]. It primarily affects the elderly, with a prevalence of up to 25 % in individuals over 85 years old [3], making it the second most common bone disease in Western countries, after osteoporosis [4], with a particularly high prevalence in Western Europe [5]. While the pelvis, proximal femur, and spine are most commonly affected, the tibia is also frequently involved, although to a lesser extent [5].

Despite its relatively high prevalence, the management of fractures in PDB remains poorly defined in the literature, with a lack of treatment guidelines, particularly in cases involving periprosthetic or proximal tibial fractures in geriatric patients. These cases are particularly challenging due to altered bone architecture, high porosity, and increased risk of delayed healing or fixation failure.

We present the case of a 91-year-old male who sustained a periprosthetic proximal tibial shaft fracture with preexisting Paget's disease of the tibia and prior TKA. We discuss the diagnostic and therapeutic considerations, surgical approach, and clinical outcome.

## Case report

**Fig. 1:**
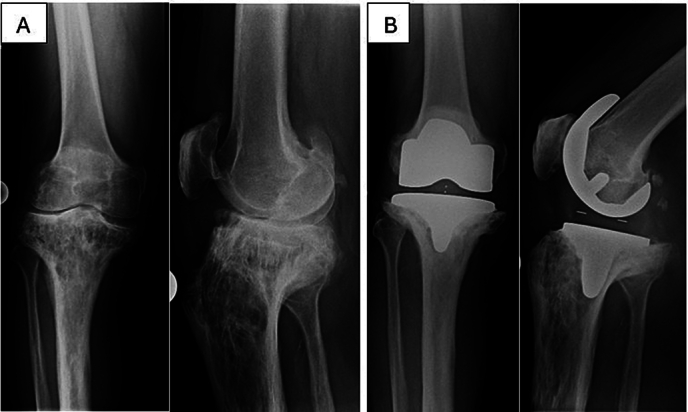
A Anteroposterior (AP) and lateral radiographs of the right knee from 2013 showing Paget's disease of the proximal tibia prior to TKA. B Postoperative AP and lateral radiographs of the right knee following TKA in 2013.

A 91-year-old male presented to the emergency room in June 2024 with severe pain in his right knee and an inability to bear weight after falling down the stairs and hitting his right knee. His medical history revealed a cement-free TKA performed in 2013 for advanced tricompartmental osteoarthrosis, atrial fibrillation requiring oral anticoagulation, and stable chronic renal failure. Radiographs from 2013, taken before and after the TKA, already displayed distinctive features of PDB of the proximal tibia [Fig. 1 A, B], which remained medically untreated. Before the injury, the patient was independently mobile without a walking aid.

### Clinical findings

The clinical examination revealed pronounced swelling due to a hematoma, tenderness and crepitus over the proximal tibia, and visible axis deviation. He experienced significant pain-related impairment in active flexion and extension. Neurovascular examination of the foot was unremarkable, with no signs of compartment syndrome.

### Diagnostic assessment

**Fig. 2:**
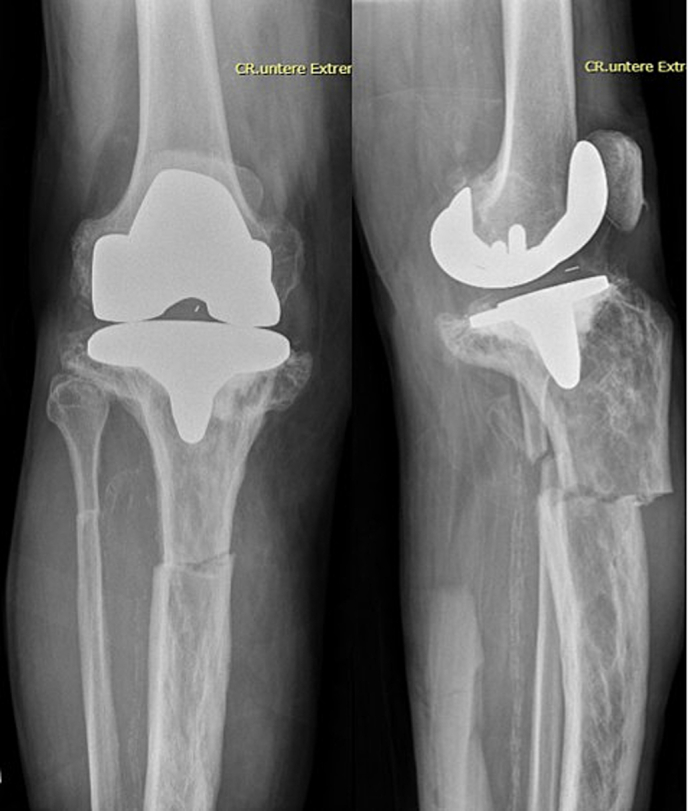
Right knee AP radiograph showing a horizontal fracture of the proximal tibial shaft, and right knee lateral radiograph revealing the fracture with an axial deviation of 9°.

**Fig. 3:**
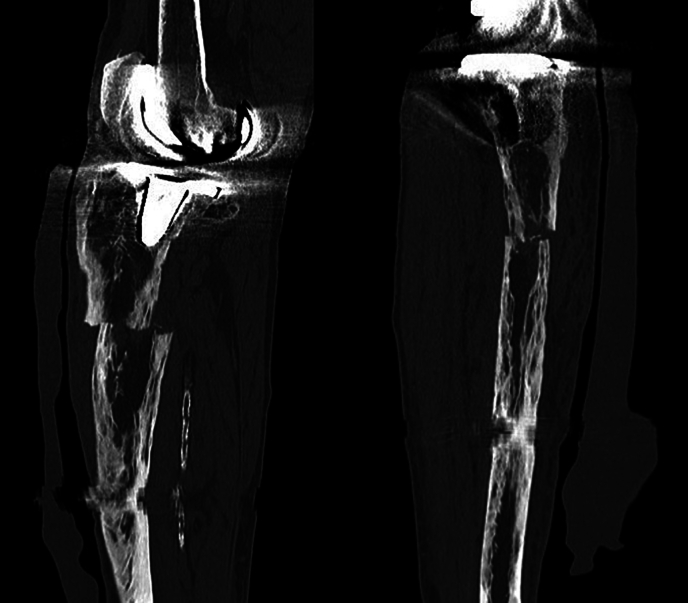
Sagittal and coronal CT scan planes showing diffuse osteosclerotic and osteolytic changes in the proximal tibia and tibial shaft.

Conventional radiographs in two planes of the right knee and lower leg revealed a dorsally dislocated, horizontal periprosthetic proximal tibial shaft fracture without prosthesis loosening but a complete deformity of the tibial plateau [Fig. 2]. Additionally, a horizontal, anteriorly dislocated proximal fibular fracture was identified. According to the Felix classification, this represents a Type III periprosthetic fracture [6]. The radiographs also showed a heterogeneous mixture of osteosclerotic and osteolytic bone texture. The CT scan later confirmed the findings by revealing cortical thickening, bone enlargement, and trabecular coarsening, all consistent with PDB [Fig. 3]. A follow-up radiograph taken three days later revealed a secondary dislocation of the tibial fracture with an increased axial deviation from 9° to 20°.

### Therapeutic intervention

**Fig. 4:**
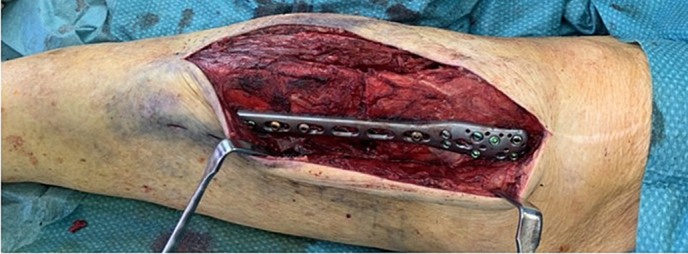
Intraoperative image showing the PHILOS plate fixed to the proximal tibia and tibial shaft after bone cement insertion and fracture reduction.

**Fig. 5:**
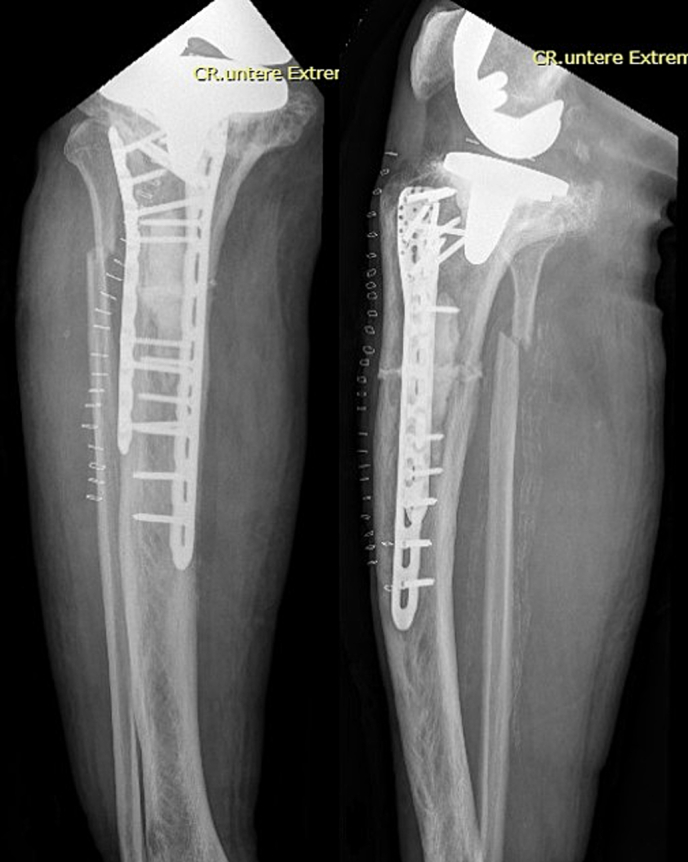
Postoperative AP and lateral radiographs of the right knee and lower leg showing the PHILOS and LCP plates in situ.

The anatomy of the proximal tibia, due to the bony deformation, made it impossible to use a standard proximal tibial plate; therefore, we indicated an open reduction and double-plate compound osteosynthesis with a PHILOS and LCP plate. Surgery was performed through an anterolateral approach, extending the pre-existing scar laterally. Intraoperatively, the bone showed signs of advanced remodeling and osteolysis. Bone samples were taken for histological analysis. After open reduction, an 8-hole 3.5 mm PHILOS plate was positioned medially to conform to the bone's contour. Next, polymethyl methacrylate bone cement was inserted into the osteolytic areas at the proximal and distal fracture ends before the PHILOS plate was definitively secured [Fig. 4]. Finally, a 10-hole 3.5 mm LCP plate was attached laterally to the proximal tibia. The fibular fracture was left untreated, given its stable alignment and minimal displacement. Standard perioperative antibiotic prophylaxis was administered. Postoperative imaging confirmed satisfactory fracture reduction [Fig. 5].

### Follow-up and outcomes

**Fig. 6:**
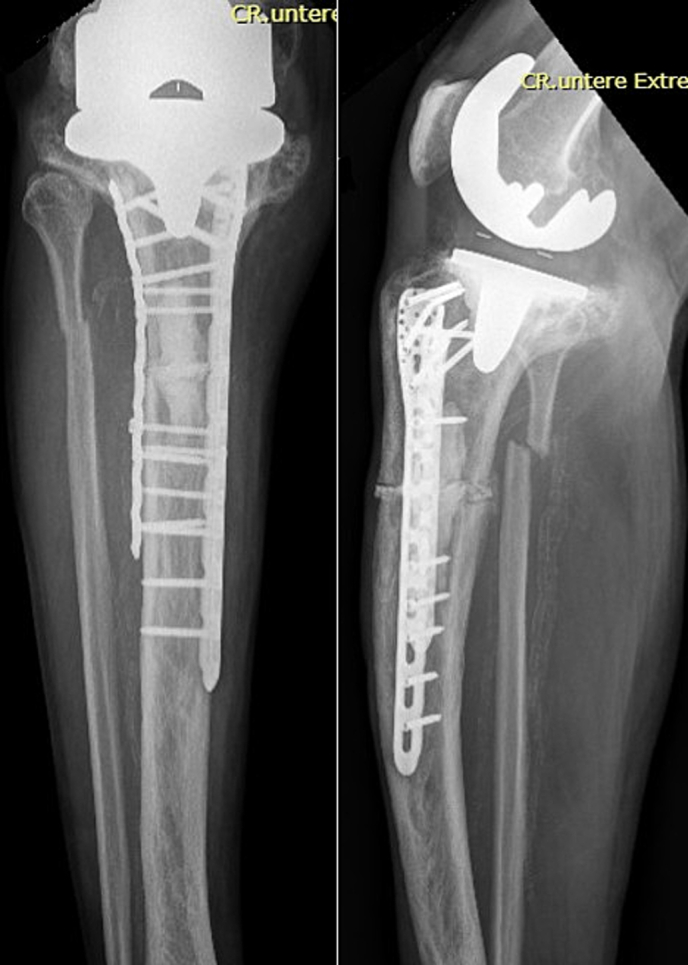
Six-week postoperative AP and lateral radiographs of the right knee and lower leg demonstrate early signs of fracture consolidation without signs of hardware loosening.

Histological analysis confirmed the diagnosis of PDB. However, bisphosphonate therapy was not initiated given the patient's advanced age, long-standing untreated disease, and absence of chronic symptoms. The patient began mobilization with physiotherapy on the first postoperative day and was allowed full weight-bearing without the need for a knee brace. He recovered quickly and was discharged two days postoperatively, once he was able to fully mobilize independently. At discharge, the patient had an active flexion of 110° and full extension (0°). At the 6-week follow-up, he demonstrated good wound healing, a pain-free knee with a range of motion from 0° to 130° and was fully weight-bearing using a forearm walker. Radiographs showed stable positioning of the fracture with the double plating system in place, with no signs of hardware loosening or migration [Fig. 6].

Unfortunately, the patient passed away due to natural causes shortly before the one-year follow-up. Until that time, no postoperative complications had occurred.

## Discussion

PDB is a chronic bone disease of unknown etiology. It is often asymptomatic and diagnosed incidentally or after complications such as fractures or secondary osteoarthritis, which may require joint replacement, as in this case. Diagnostic modalities include laboratory findings such as elevated alkaline phosphate levels, radiographs, and CT scans, which can be supported by histological analysis. The treatment of PDB focuses on symptom management and the prevention of complications, primarily through bisphosphonates [7], but surgical intervention becomes necessary in cases of severe arthritis or fractures.

While few studies address fracture management in PDB, only one case report to date specifically describes surgical treatment of a tibial fracture in a PDB patient [8], highlighting both the rarity of such injuries and the challenges of surgical intervention due to compromised bone quality. To our knowledge, no other reports exist on the surgical treatment of tibial shaft fractures with underlying PDB.

Tibial shaft fractures, the most common long bone fractures, occur at an incidence of 16.9 per 100,000 individuals per year [9]. They are typically associated with low energy falls in elderly individuals. These fractures are linked to considerable short- and long-term morbidities, including acute compartment syndrome and extensive soft tissue injury, and to chronic leg and knee pain [10]. Proximal tibial shaft fractures present additional challenges due to their proximity to key anatomical structures, such as the patellar tendon, iliotibial band, and the pes anserinus. When using an anterolateral approach as we did, injury to the superficial peroneal nerve must be avoided.

In cases of prior TKA, implant stability must be assessed first to rule out prosthesis loosening, as this impacts surgical decision-making. Currently, there are no strict guidelines for treating periprosthetic proximal tibial shaft fractures. Some studies suggest closed reduction and intramedullary nailing for fractures distal to the prosthesis [11], a technique requiring high surgical skills, while others recommend open reduction and internal fixation [12]. Given the variability of these fractures due to factors like fracture location, bone quality, and patient comorbidities, an individualized approach is essential for optimal treatment.

In our case, the decision-making was influenced by both the unstable, secondarily dislocated fracture and the presence of underlying PDB. Considering these factors, we opted for open reduction with double plating, along with the use of bone cement for additional support, as it is recommended for pathological fractures due to its high mechanical strength and durability. This approach enhanced stability and rigidity by providing structural support, allowing immediate weight-bearing, and therefore reducing postoperative complications. To achieve greater stability in highly porous bone, we used a PHILOS plate alongside an LCP, providing enhanced fixation while also conforming better to the proximal tibial anatomy. As the incidence of periprosthetic fractures is projected to rise by 2.5 times, with associated costs increasing tenfold over the next decade [13], optimizing management strategies becomes crucial to reduce both the clinical and economic burden on healthcare systems.

Nonetheless, even with appropriate surgical management, tibial shaft fractures carry a considerable risk of complications such as malunion and nonunion, with nonunion rates of up to 7.4 % [14]. These risks are further elevated in proximal tibial fractures involving the metaphysis, which are prone to valgus deformity, and in PDB patients, where preexisting bowing makes anatomical alignment particularly challenging, as both overcorrection and inadequate reduction must be avoided. Horizontal fracture patterns, such as in our case, are additionally known for poor healing tendencies. PDB itself is associated with impaired fracture healing, often leading to delayed union or nonunion after surgical fixation [15]. Due to the patient's death shortly before the one-year follow-up, we are unable to assess the long-term outcome in terms of bone healing, malunion, or nonunion, which constitutes a limitation of our case report.

In elderly patients with periprosthetic fractures, achieving stable fixation is essential to enable early or immediate full weight-bearing, as prolonged immobility can result in severe complications. Recovery is generally slower in patients over 40 years [16], contributing to increased morbidity, mortality, extended hospital stays, and higher healthcare costs. Geriatric patients are particularly vulnerable, often losing their previous level of independence. Since partial weight-bearing is difficult for many, achieving solid osteosynthesis becomes essential to facilitate early mobilization and functional recovery.

## Conclusion

We report a rare case of a periprosthetic proximal tibial shaft fracture in a patient with underlying PDB. Due to the compromised bone quality and complex fracture pattern, an individualized surgical approach using double-plate compound osteosynthesis was essential. This provided immediate postoperative stability, enabled full weight-bearing, and allowed fast rehabilitation, thereby reducing postoperative complications and shortening hospitalization time. Periprosthetic fractures in PDB pose a unique diagnostic and therapeutic challenge, particularly in elderly patients. Conventional fixation methods may be insufficient due to advanced bone deformation and high porosity. Unconventional techniques, such as using a PHILOS plate for the medial proximal tibia in combination with bone cement insertion, can improve stability and lead to good clinical outcomes. This case report highlights the need for larger studies on the management of PDB-related fractures of the lower extremities, particularly in elderly patients or in the setting of periprosthetic fractures, to establish more standardized treatment guidelines.

## CRediT authorship contribution statement

**Melinda Schlink:** Writing – original draft, Conceptualization, Investigation, Methodology, Visualization. **Christian von der Lippe:** Writing – review & editing, Supervision.

## Patient consent

Written informed consent was obtained from the patient for publication of this case report and accompanying images prior to his death.

## Acknowledgements and funding

This research did not receive any specific grant from funding agencies in the public, commercial, or not-for-profit sectors.

## Declaration of competing interest

The authors declare that they have no competing interests.
